# Role of Tumor Associated Fibroblasts in Human Liver Regeneration, Cirrhosis, and Cancer

**DOI:** 10.4061/2011/120925

**Published:** 2011-12-01

**Authors:** Daniela Cesselli, Antonio Paolo Beltrami, Alessandra Poz, Stefania Marzinotto, Elisa Comisso, Natascha Bergamin, Evgenia Bourkoula, Anja Pucer, Elisa Puppato, Barbara Toffoletto, Marisa Sorrentino, Umberto Baccarani, Claudio Avellini, Carlo Alberto Beltrami

**Affiliations:** ^1^Department of Medical and Biological Sciences, University of Udine, P.le Kolbe 3, 33100 Udine, Italy; ^2^Department of Pathology, University Hospital “S. Maria della Misericordia” of Udine, Piazzale Santa Maria della Misericordia 15, 33100 Udine, Italy; ^3^University of Ferrara and Cardiovascular Research Center, Salvatore Maugeri Foundation, IRCCS, 44100 Ferrara, Italy; ^4^Department of Surgery, University Hospital “S. Maria della Misericordia” of Udine, Piazzale Santa Maria della Misericordia 15, 33100 Udine, Italy

## Abstract

Tumor associated fibroblasts (TAFs) are considered a microenvironmental element critical for tumor growth and progression. Experimental studies suggest that their origin could be from mesenchymal stem cells (MSCs) derived from the bone marrow. However, the role played by TAFs in cirrhosis, hepatocellular carcinoma development, and progression is largely unknown, and *in vitro* human models are missing. This paper for the first time demonstrates that (1) human neoplastic livers possess a population of multipotent adult stem cells (MASCs) with properties of TAFs; (2) a population of MASC-derived TAFs is already present in cirrhotic, not yet neoplastic, livers; (3) MASCs isolated from nonneoplastic and noncirrhotic liver scan acquire a TAF phenotype when grown in a medium conditioned by tumor cell lines, supporting the notion that TAF could originate from resident primitive cells (MASCs), possibly through a paracrine mechanism.

## 1. Introduction

Recently, it has been recognized the existence, in the liver, of a strict connection linking development, regeneration, and carcinogenesis [1, 2]. As a consequence, researchers are trying to dissect the molecular mechanisms regulating liver homeostasis, the comprehension of which could open the way to new targeted therapies for liver regeneration, liver cirrhosis, and primary liver cancers [1, 2].

In this regard, during liver injury, the reparative processes take advantage of two principal strategies of defense, depending on the extent of the damage [1]. While mild injury is mainly repaired through compensatory hyperplasia of hepatocytes, severe damage implies the activation of a liver progenitor cell compartment [2]. In both cases, other nonparenchymal cells (stellate cells, vascular, and biliary endothelial cells) proliferate as soon as hepatocytes start to [2] and cooperate to restore morphofunctional competent liver tissue. Conversely, chronic liver diseases are often characterized by an unbalance between epithelial cell proliferation and stroma remodeling. As a consequence, the clinical course of chronic liver diseases significantly depends on the progression rate and the extent of fibrosis [3]. This latter may be considered as an unrestricted wound healing process, which causes matrix synthesis secondary to the activation of hepatic stellate cells, fibroblasts, hepatocytes, and biliary epithelial cells. These latter two are converted to matrix-producing (myo-) fibroblasts by a process defined as epithelial-mesenchymal transition [3, 4].

Epidemiologically, chronic wounds and inflammatory states are well-known risk factors for cancer development and the connection between cirrhosis and liver cancer is paradigmatic [5, 6]. In this regard, it has been proposed that tumor stroma can be considered as “normal wound healing gone awry [7],” able to interact through paracrine and juxtacrine pathways with the tumor stem cells that are influenced by the microenvironment in which they are endowed [8–12]. Cells of the tumor microenvironment, therefore, play active roles in determining the malignant phenotype [12–16]. 

Among the constituents of the tumor microenvironment, a key role in tumor progression, tumor growth, angiogenesis, and metastasis is played by the tumor associated fibroblast (TAF), also named cancer-associated fibroblasts (CAFs), [11, 12, 16–18]. TAFs are characterized by the expression of specific markers [9, 18–22] and, although their origin remains controversial, their presence may be essential for the invasive growth of the tumor, which culminates into a poor clinical prognosis [12, 18]. Thus, management of stromal reaction through prevention of the formation of TAFs is a potentially attractive opportunity for therapy [17].

Regarding liver cancer, although it has been demonstrated the presence of activated myofibroblasts, possibly originating from activated hepatic stellate cells [23], the role played by TAFs in cirrhosis, HCC development, and progression is largely unknown, and *in vitro* human models are missing.

Recently, our group has optimized a method to isolate, from several human adult tissues, a population of primitive cells named multipotent adult stem cells (MASCs), characterized by a mesenchymal immunophenotype, clonogenicity, and a wide *in vitro* differentiation capacity, being able, independently from the tissue of origin, to give rise to multiple mature cell types of all the three germ layers [24, 25]. 

In this work, we decided to investigate the possibility to isolate and characterize MASCs both from neoplastic and from cirrhotic human liver tissues to test the hypothesis that liver-resident MASCs could generate TAFs in pathological conditions such as cirrhosis or liver cancer. Interestingly, we demonstrated the existence of a population of MASCs characterized by TAFs characteristics both in human hepatocellular carcinoma and in cirrhotic livers, suggesting that MASCs with TAF features can contribute to the development of a microenvironment favoring HCC onset. To further support the hypothesis that MASCs obtained from normal livers could generate TAFs, when exposed to HCC cells, first we demonstrated that MASCs isolated from nonneoplastic, non-cirrhotic livers do not possess aberrant growth properties, then we showed that they can acquire TAFs features when exposed to specific inducing conditions, supporting for the first time the notion that TAFs can originate from resident primitive cells.

## 2. Materials and Methods

Human liver samples were collected by the Surgery Department of the Azienda Ospedaliero Universitaria of Udine, after informed consent was obtained, in accordance with the Declaration of Helsinki, and with approval by the Independent Ethics Committee of the University-Hospital of Udine. 

### 2.1. Tissue Donors

The principal characteristics of tissue donors are summarized in Table 1. Samples were obtained from (1) nonneoplastic and noncirrhotic livers discarded for orthotopic liver transplantation for clinical reasons; the histological conditions of the livers showed the presence of a moderate grade of steatosis, mainly microvesicular, without fibrosis of the portal trees or necrosis of the hepatocytes (L); (2) cirrhotic livers affected by hepatocellular carcinoma (HCC); and (3) cirrhotic livers not affected by HCC. In particular, in HCC livers we obtained samples from two different regions: the neoplastic area (N) and a remote, cirrhotic, macroscopically nonneoplastic region (C). While half of each sample was formalin fixed-paraffin embedded and microscopically evaluated by an expert pathologist, the other half was utilized for cell isolation.

### 2.2. MASCs Isolation and Culture

Cells from nonneoplastic and noncirrhotic livers were isolated and cultured as described in [24]. Liver sample fragments obtained from cirrhotic and neoplastic livers were first disaggregated mechanically with scalpels and then enzimatically dissociated in a 0.25% collagenase type IV solution (Worthington) in Joklik modified Eagle's Medium (Sigma-Aldrich) for 5 minutes at 37°C. Cell suspension was filtered through a sieve (BD Falcon) in order to select a population less than 40 *μ*m in diameter.

However 2 × 10^6^ freshly isolated human cells were plated onto 100 mm human fibronectin- (Sigma-Aldrich) coated dishes (BD Falcon) in an expansion medium previously described [24]. Once cells reached 70-80% of confluence, they were detached with 0.25% trypsin-EDTA (Sigma-Aldrich) and replated at a density of 1-2 × 10^3^/cm^2^. Cells obtained from nonneoplastic noncirrhotic livers were named L-MASCs, while those obtained from HCC and cirrhotic specimens were named N- and C-MASCs, respectively.

### 2.3. Cell Growth Kinetic

Cells were seeded at a density of 2,000 cells/cm^2^ in expansion medium. Cells were detached and counted at 1, 2, 5, 9, 12, and 14 days.

### 2.4. Induction of *In Vitro* Multilineage Differentiation

L-, N- and C-MASCs were differentiated using the media described in [24]. At the end of every treatment, cells were fixed either with 4% buffered paraformaldehyde or with a solution of absolute ethanol: 40% formalin, 9 : 1.

### 2.5. Functional Assays

Functionally competent myocytes were detected by the presence of spontaneous calcium transients, as evaluated by Fluo-4 imaging (Molecular Probe, Invitrogen). Fluo-4/DMSO stock solution was diluted to a final concentration of 1–5 *μ*M in a buffered physiological medium. The nonionic detergent Pluronic, at a final concentration of 0.02%, was added. Cells were incubated with Fluo-4 solution at 37°C and, after 20 minutes, washed in indicator-free medium. Image acquisitions were performed using a Leica DMI 6000B inverted microscope. Images were acquired at a 400x magnification (HCX PL Fluotar objective), collecting one image in both Fluo-4 and phase contrast channels every 5.6 seconds for a period of 90 seconds.

Active calcium deposition sites were identified by tetracycline (Sigma-Aldrich) incorporation into osteogenic areas of osteogenic differentiating cultures, as described in [24]. 

The active uptake of acetylated low-density lipoprotein (LDL), the presence of glycogen by periodic acid Shiff (PAS) staining, and PAS after diastase digestion were used as markers of hepatocyte function. To test the *in vivo *uptake of DiI-labeled acetylated LDL (Molecular Probes), cells were incubated for 4 hours at 37° with 15 *μ*g/mL acetylated LDL and cell fluorescence was analyzed by Leica DMI 6000B microscope. For PAS staining samples were fixed in a solution of absolute ethanol: 40% formalin 9 : 1 for 4 minutes, incubated in 1% periodic acid, and then incubated with Schiff's reagent for 30 minutes in darkness and counterstained with hematoxylin for 10 minutes. Diastase digestion was performed by treating section with 1 *μ*g/mL preheated diastase solution (Sigma-Aldrich) at 37°C for 15 minutes.

### 2.6. Soft Agar Assay

To evaluate the ability of L-, N-, and C- MASCs to grow in an anchorage-independent way, 50,000 cells were plated in a 0.25% soft agar solution in 35 mm plates containing a basal layer of 1% agarose; colonies with a diameter greater than 100 microns were counted after 4 weeks under a phase contrast microscope (Leica DMI 6000B). For the comparison of L-MASCs cultured in normal medium and in semiconditioned medium obtained from HepG2 [26] and HuH7 [27] cell lines, colony formation was analyzed 2 weeks after seeding. For this purpose, a series of optical sections, obtained by scanning along the *z*-axis: the field of interest, were acquired at a 100x magnification, using the Leica DMI-6000B setup. Once obtained the sum image, the number of colonies with a diameter greater than 60 microns was quantified. For each replicate, a volume of 500 *μ*m^3^ was sampled.

### 2.7. Conditioning of L-MASCs with Semiconditioned Medium (CM) from HepG2 and HuH7

The HCC cell lines HepG2 [26] and HuH7 [27] were used. One day after seeding into T75 flasks (2 × 10^6^ cells), HepG2 and HuH7 were washed twice with serum-free HBSS (Sigma-Aldrich), and then incubated for 24 hours with serum-free MASCs proliferation medium (15 mL/T75, Sigma-Aldrich). Medium was then collected, filtered through a 0.22 *μ*m filter (Millipore), and kept at +4°C. Serum-free MASCs proliferation medium incubated for 24 hours in cell culture flasks without cells was used as control. 

For conditioning experiments, conditioned media obtained from HepG2, HuH7 and empty flask, were diluted 1 : 1 with MASCs proliferation medium in order to obtain HepG2-CM, HuH7-CM and non-CM, respectively.

However L-MASCs (*n* = 3) were cultured in HuH7-, HepG2- and non-CM for 3 passages (*≈*12 population doublings) and finally analyzed in terms of population doubling time, surface immunophenotype, pluripotent-state specific transcription factor expression and assayed in migration and soft-agar assays. All experiments were performed in triplicate.

### 2.8. Scratch Assay

In order to evaluate *in vitro* cell migration of L-MASCs conditioned, or not, by HepG2 and Huh7, a scratch assay was performed [28]. In 33 mm plate at high confluence, scratches were created utilizing 200 *μ*L tips. Phase contrast images of the scratches were acquired at 3-hour intervals, until their complete closing, utilizing Leica DMI 6000B. Images were then compared and quantified by Image J in order to calculate the rate of cell migration. The mean scratch width did not differ significantly in the three culture conditions being of 553 ± 51 *μ*m, 555 ± 131 *μ*m, and 515 ± 72 *μ*m in non-CM, HepG2-CM, and Huh7-CM, respectively (*P* > .05).

### 2.9. Reverse Transcriptase PCR Analysis

Total RNA was extracted from nonconfluent cultures of N- and C-MASCs at P3 or L-MASCs cultured in non-CM, HepG2-CM, and Huh7-CM using the TRIzol Reagent (Invitrogen). After treatment with DNase I (Ambion), first strand cDNA synthesis was performed with 1 *μ*g total RNA using random hexanucleotides and MMLV reverse transcriptase (Invitrogen). PCR amplification was carried out in a final volume of 50 *μ*L, using 80–150 ng cDNA, 10 mM Tris-HCl pH 9.0, 1.5 mM MgCl_2_, 0.2 mM dNTPs, 25 pmol of each primer, and 2 U Taq I polymerase (Amersham). The PCR conditions were as follows: 94°C for 2 minutes; 40 cycles at 95°C for 30 seconds, 61°C for 60 seconds. and 72°C for 60 seconds. The optimal conditions and the number of cycles were determined to allow amplification of samples within the linear phase of the PCR. The reaction products were analyzed on 3% agarose gels. Primer pairs and product length were the following: OCT4 reference sequence ENST00000259915 primer FW 5′CGAAAGAGAAAGCGAACCAGTAT3′ and primer RW 5′CGAGAGGATTTTGAGGCTGCT3′ (product lenght 216 bp); SOX2 (SRY-box2) reference sequence ENST00000325404 primer FW 5′ATGGGTTCGGTGGTCAAGT3′ and primer RW 5′CCTGTGGTTACCTCTTCCTCC3′ (product length 60 bp); NANOG reference sequence ENST00000229307 primer FW 5′ATGCCTCACACGGAGACTGT3′ and primer RW 5′TGCTTATTCAGGACAGCCCT3′ (product length 66 bp); GAPDH (glyceraldehyde-3-phosphate dehydrogenase) reference sequence ENST00000229239 primer FW 5′ACCCACTCCTCCACCTTTGACG3′ and primer RW 5′AGCAAGAGCACAAGAGGAAGAGAGA′3 (product length 187 bp); ACTA2 (actin, *α*2, smooth muscle) reference sequence ENSG00000107796 primer FW 5′CTGTTCCAGCCATCCTTCAT3′ and primer RW 5′ACAGGAATACGATGAAGCCG3′ (product lengh 316 pb); THSBS1 (Thrombospondin1) reference sequence ENSG00000137801 primer FW 5′TCAGGACCCATCTATGATAAAACCTA3′ primer RW 5′GAAATGGTGTTCTTCTCTGA3′ (product lengh 85 pb); FAP (fibroblast activation protein) reference sequence ENSG0000078098, primer FW 5′ATCTATGACCTTAGCAATGGAGAATTTGT3′ and primer RW 5′GTTGGGAGTAAATTAGCATATGTCTATCAAAAC3′ (product lengh 171 pb); FSP1 (S100A4) reference sequence ENSG00000196154, primer FW 5′TCTTTCTTGGTTTGATCCTG3′ and primer RW 5′TTCAGACACGTGCTTGATGC3′ (product lengh 450 pb). 

### 2.10. Flow-Cytometry

Proliferating cells were detached with 0.25% trypsin-EDTA (Sigma-Aldrich) and, after a 20-minute recovery phase, were incubated with either properly conjugated primary antibodies: CD13, CD29, CD49a, CD49b, CD49d, CD90, CD73, CD44, CD59, CD45, HLA-DR, CD117, CD271, CD34, (BD Biosciences), CD105, CD66e (Serotech), CD133 (Miltenyi Biotec), E-cadherin (Santa Cruz Biotechnology), ABCG-2 (Chemicon International), or with an unconjugated primary antibody: N-cadherin (Sigma-Aldrich). Unconjugated antibody was revealed using PE- or FITC-conjugated secondary antibodies (DakoCytomation). Properly conjugated isotype-matched antibodies were used as a negative control. The analysis was performed either by FACS-Calibur (BD Biosciences) or by CyAn (Dako Cytomation).

### 2.11. Immunofluorescence and Histochemistry

Cells cultured either in expansion or in differentiation medium were fixed in 4% buffered paraformaldehyde for 20 minutes at room temperature (RT). For intracellular stainings, fixed cells were permeabilized for 8 minutes at RT, with 0.1% Triton X-100 (Sigma-Aldrich) before exposing them to primary antibodies. Primary antibody incubation was performed over night at 4°C using the following dilutions: Oct-4 (Abcam, 1 : 150); Sox-2 (Chemicon, 1 : 150); Nanog (Abcam, 1 : 150); Cytokeratins 7, 8, 18, 19 (Biogenex, 1 : 20); *β*3-tubulin (Abcam, 1 : 1000); Smooth Muscle Actin (Dako, 1 : 50); Connexin 43 (Santa Cruz, 1 : 40); *α*-Sarcomeric Actin (Sigma, 1 : 100); Osteocalcin (Abcam, 1 : 100); Gata4 (Santa Cruz, 1 : 100). For the following antibodies the incubation was performed one hour at room temperature: Vimentin (Dako 1 : 500), Desmin (Dako 1 : 200), Tenascin-C (Novocastra 1 : 200), and VEGF (Neomarkers 1 : 100). To detect primary antibodies, A488 and A555 dyes labeled secondary antibodies, diluted 1 : 800, were employed (Molecular Probe, Invitrogen). Finally, 0.1 *μ*g/mL DAPI (Sigma) was used to identify nuclei. Vectashield (Vector) was used as mounting medium. 

Alcyan blue staining was performed in cells fixed in a solution of absolute ethanol: 40% formalin (9 : 1). After 1 minute of incubation in Alcyan blue at pH 1.0, cells were washed in HCl 0.1 N and counterstained with nuclear-fast Red. 

von Kossa stain was performed in 4% paraformaldehyde fixed cells, treated with 5% silver nitrate solution in front of a 60-watt lamp for 1 hour, and incubated in 5% sodium thiosulphate. Cells were counterstained with nuclear-fast Red.

Confocal image acquisition was carried out by a Confocal Laser Microscope (Leica TCS-SP2, Leica Microsystems) utilizing either a 63x oil immersion objective (numerical aperture: 1.40) or a 40x oil immersion objective (numerical aperture: 1.25). Epifluorescence and phase contrast images were obtained utilizing a live cell imaging dedicated system consisting of a Leica DMI 6000B microscope connected to a Leica DFC350FX camera (Leica Microsystems, Wetzlar, Germany). Also, 10x (numerical aperture: 0.25), 40x oil immersion (numerical aperture: 1.25), and 63x oil immersion (numerical aperture: 1.40) objectives were employed for this purpose. Bright field images were captured utilizing a Leica DMD108 microscope (Leica Microsystems). Moreover, 10x (numerical aperture: 0.40), 20x (numerical aperture: 0.70), and 40x (numerical aperture: 0.95) objectives were employed. Adobe Photoshop software was utilized to compose, overlay the images and adjust the contrast (Adobe, USA).

### 2.12. Statistics

Two-tailed unpaired-Student *t*-test and one-way Anova followed by Bonferroni posttest were utilized to compare means between two or more groups, respectively (Prism, version 4.0c). Results are expressed as mean ± SD. *P* values less than  .05 were considered significant.

## 3. Results

### 3.1. Multipotent Adult Stem Cells (MASCs) Can Be Obtained from Neoplastic and Cirrhotic Livers

In order to establish whether human cirrhotic livers possess a population of multipotent adult stem cells (hMASCs), we applied the method, previously optimized to isolate hMASCs from non-neoplastic, noncirrhotic livers [24], to *n* = 15 livers affected by HCC. Cell lines were obtained both from HCC areas (N) and from cirrhotic areas distant from HCC (C), which were macroscopically and microscopically devoid of neoplastic foci. We named the cell lines N-MASCs and C-MASCs, respectively. MASCs were also isolated from *n* = 3 specimens obtained from cirrhotic livers not affected by HCC. As a control, we utilized *n* = 5 cell lines obtained from nonneoplastic, noncirrhotic controls [24] (L-MASCs). The clinical features of tissue donors analyzed in this study are summarized in Table 1. 

In line with the epidemiological data, most of the patients were males (93%). All the patients but 1 were affected by a chronic viral hepatopathy and 87% underwent transarterial chemoembolization (TACE) before surgery. Surgical treatment consisted in orthotopic liver transplantation in 87% of cases. About half of neoplasias were at low grade (G1 and G2).

**Figure 1:**
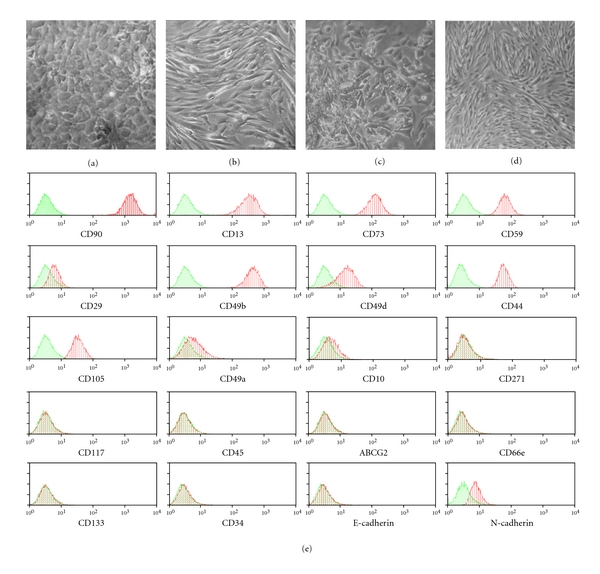
(a–d) Phase contrast images of C-MASCs (a, b) and N-MASCs (c, d) in primary culture (a–c) and at the third passage in culture (b, d). (e) FACS analysis of N-MASCs. Representative flow cytometry histograms of multipotent cell populations. Plots show isotype control IgG-staining profile (green histogram) versus specific antibody staining (red histogram).

After 15–20 days from the primary culture, polymorphic colonies were detected in about 60% and 80% of the N and C samples, respectively (Figures  1(a)–1(d)). As previously described, MASCs were obtained with a 100% efficiency from nonneoplastic, noncirrhotic livers. Differences in the isolation method (digestion in perfusion versus mechanical-enzymatic digestion), in the composition of the tissue and/or in the frequency of cells able to grow in culture can explain the lower efficiency of the method in isolating MASCs from cirrhotic samples. TACE, performed before surgery, could also have interfered with the lower efficiency in isolating MASCs, especially from the neoplastic region.

After three to four passages in expansion medium, MASCs displayed a homogeneous, fibroblast-like morphology, independently from the tissue of origin (Figures  1(b)–1(d)). With respect to L-MASCs, C- and N-MASCs were characterized by a significantly longer population doubling time (27 ± 2, 49 ± 7, and 58 ± 9 hours, resp.; *P* < .05 versus L-MASCs). However, despite the differences in growth kinetic, N-, C-, and L-MASCs behaved as finite cell lines able to proliferate for more than 40 population doublings before reaching cell senescence and growth arrest.

### 3.2. Multipotent Adult Stem Cells (MASCs) Obtained Either from Neoplastic or Cirrhotic Livers Possess Stem Cell Features and Aberrant Growth Properties

MASCs were studied in terms of surface immunophenotype, expression of pluripotent state-specific transcription factors, and multipotency.

Considering the surface immunophenotype, as evaluated by flow-cytometry, N-, C-, and L-MASCs shared a similar mesenchymal immunophenotype (Figure  1(e) and Table 2). Interestingly, while N- and C-MASCs did not differ in the expression of any of the tested markers, some differences were seen between these latter and L-MASCs (Table 2). Specifically, L-MASCs, with respect to N- and C-MASCs, were characterized by a lower expression of *α*(2)-integrin (CD49b) and by a higher expression of *α*(4)-integrin (CD49d) (Table 2). 

The role of integrins in tumor growth and metastasis is well established [29]. Interestingly, *α*(2)-integrin has been described to play a crucial role in hepatocarcinoma cell invasion and metastasis [30], while a transcriptional repression of the integrin *α*4 gene caused by aberrant DNA methylation has been demonstrated in gastric cancer and cholangiocarcinoma [31]. 

**Figure 2:**
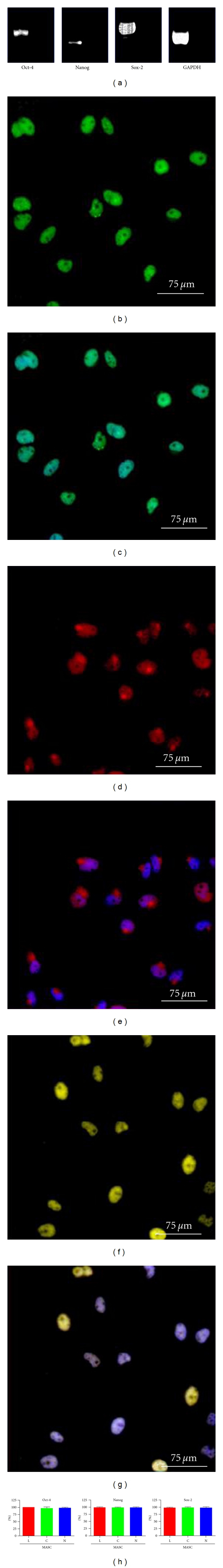
Pluripotent state-specific transcription factor expression. (a) Representative RT-PCR analysis of Oct-4, Sox-2, Nanog, and GAPDH mRNA transcripts in N-MASCs. The right lane of each gel is the negative control (H_2_O). (b–g) Oct-4 (green fluorescence; (b–c)), nanog (red fluorescence; (d, e)), and sox-2 expression (yellow fluorescence; and (f, g)) in the nuclei of C-MASCs. Nuclei are depicted by the blue fluorescence of DAPI staining (c, e, g). (h) Quantification of pluripotent state-specific transcription factor expression. Data are presented as mean ± standard deviation.

Regarding the expression of Oct-4, Nanog, and Sox-2, transcription factors considered to be crucial for the maintenance of embryonic stem cell self-renewal and pluripotency [32], N-, C-, and L-MASCs were shown to express them both at mRNA and protein level (Figures 2(a)–2(h)). 

In order to assess multipotency, N- and C-MASCs were induced to differentiate into multiple, mature cell types of the three germ layers and tested for the acquisition of functional as well as molecular features of differentiation [24].

**Figure 3:**
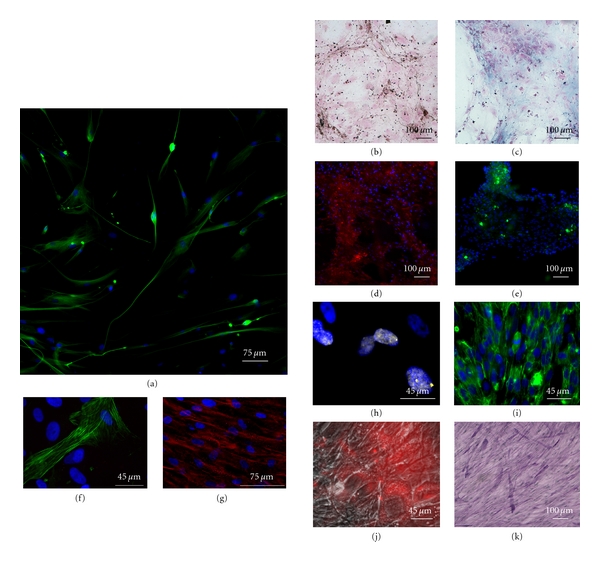
Multipotency of MASCs obtained from neoplastic livers. A neuroectodermic differentiation: N-MASCs (a) in neurogenic medium acquired the expression of *β*3-tubulin (green fluorescence). (b–e) Osteogenic differentiation: N-MASCs in osteogenic medium display positivity for von Kossa (b) and Alcyan blue (c) cytochemistry reactions, were able to incorporate tetracycline in sites of calcification when excited with a UV laser (red fluorescence, (d)) and presented osteocalcin deposits (green fluorescence, (e)). (f, g) Myocyte differentiation: a low fraction of N-MASCs in differentiation medium express the myocyte-specific filament SMA (green fluorescence, (f)), while the large part of the cells expresses *α*-sarcomeric actin in the cytoplasm (red fluorescence, (g)). (h–k) Endodermic differentiation: cells cultured for one week in a medium added with HGF and FGF-4 became positive for GATA-4 (yellow fluorescence, (h)). Cells cultured for three weeks in the same medium became positive for cytokeratins 8–18-19 (green fluorescence, (i)) were able to actively uptake DiI-labeled acetylated LDL (red fluorescence overlaid on a phase contrast image, (j)) and presented diastase-resistant glycogen granules, as revealed by PAS reaction (purple stain, (k)). In the fluorescence pictures, nuclei are depicted by the blue fluorescence of DAPI staining.

Cells exposed for three weeks to EGF and b-FGF displayed a morphological change, even if at lower extent with respect to L-MASCs. The cells became positive for *β*3-tubulin (20 ± 12%), arrayed in filaments and bundles (Figure  3(a)). 

Cells cultured in an osteogenic medium became positive both to von Kossa and Alcyan blue stainings, specific for the differentiation toward bone and cartilage, respectively (Figure  3(b) and 3(c)). Moreover, when cultured in the presence of tetracycline, a calcium chelator often used to label the mineralizing front of forming bone, N- and C-MASCs were characterized by the gradual deposition of tetracycline-labeled material, indicating that the newly formed matrix was also mineralized (Figure  3(d)), and expressed osteocalcin (Figure  3(e)). When cultured in a myogenic medium, a fraction of cells (<10%), expressed smooth-muscle actin (SMA) (Figure  3(f)), while almost all cells showed positivity for *α*-sarcomeric actin (ASA) (Figure  3(g)). The presence of functional competent receptors involved in calcium handling was demonstrated by spontaneous intracellular calcium transients, as displayed by Fluo-4 assays (Supplementary movie 1 available online at doi: 10.1155/2012/120925). 

Cells differentiated into hepatocytes became positive for the nuclear transcription factor GATA-4 (Figure  3(h)), became polygonal, and stained positive for cytokeratins 8–18-19 (Figure  3(i)). Functionally, cells acquired the abilities to actively up take acetylated LDL (Figure  3(j)) and to store glycogen, as demonstrated by PAS staining (Figure  3(k)).

Altogether, the accumulated evidences show that, similarly to what has already been demonstrated for the MASCs obtained from nonneoplastic and noncirrhotic livers [24], MASCs obtained from neoplastic and cirrhotic samples are characterized by a stem cell phenotype and by a wide *in vitro *multipotency. However, with respect to L-MASCs, N- and C-MASCs displayed an impaired plasticity, especially toward a neurogenic fate.

**Figure 4:**
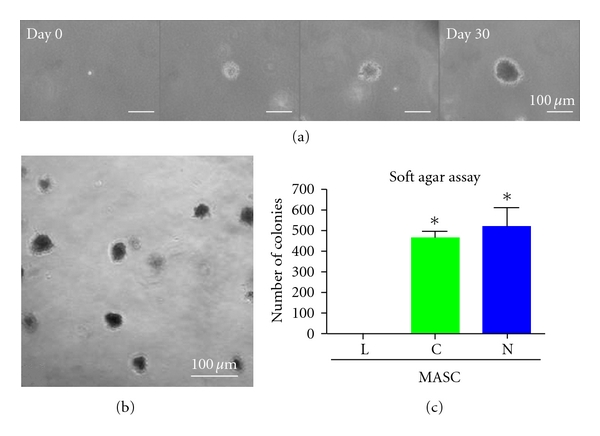
Anchorage-independent growth. Representative colonies of N-MASCs developed in soft agar at different time points (a). After 30 days, colonies were formed in N-MASCs (b) and C-MASCs culture, but not in L-MASCs culture, as represented in the histogram (c). Data are presented as mean ± standard deviation. *P < .05 versus L-MASCs.

Despite some similarities, the growth properties of N- and C-MASCs were significantly different from L-MASCs. Specifically, all MASCs derived from neoplastic and cirrhotic livers, but not those from nonneoplastic and noncirrhotic livers, developed colonies in soft agar (Figures  4(a)–4(c)), a property shared by tumorigenic cells and TAFs [33]. The ability to grow in soft agar was independent from the presence of cancer, since C-MASCs obtained from cirrhotic but nonneoplastic livers (*n* = 3) were able to grow in soft agar too (data not shown).

### 3.3. MASCs Obtained from Normal Liver Can Acquire Abnormal Growth Properties in the Presence of Neoplastic Cell Lines

In order to establish whether L-MASCs exposed to a medium conditioned by tumor cell lines (TCM) could acquire a TAF phenotype, we cultured L-MASCs (*n* = 3) for three passages in a medium conditioned by HuH7 [27] and HepG2 [26], two hepatocellular carcinoma cell lines. At the end of the treatment, L-MASCs were assessed in terms of growth kinetics, cell surface phenotype, pluripotent-state-specific transcription factor, and TAF-specific marker expression, migration ability, and adhesion-independent growth.

The population doubling time of L-MASCs did not significantly change after the exposure of cells to TCM, being of 18 ± 3, 18.5 ± 2, and 16.5 ± 2 hours in non-CM, in HepG2-CM, and in Huh7-CM, respectively (*P* > 0.05). Similarly, culture conditions did not significantly change either the surface immunophenotype or the expression of Oct-4 and Nanog (data not shown).

**Figure 5:**
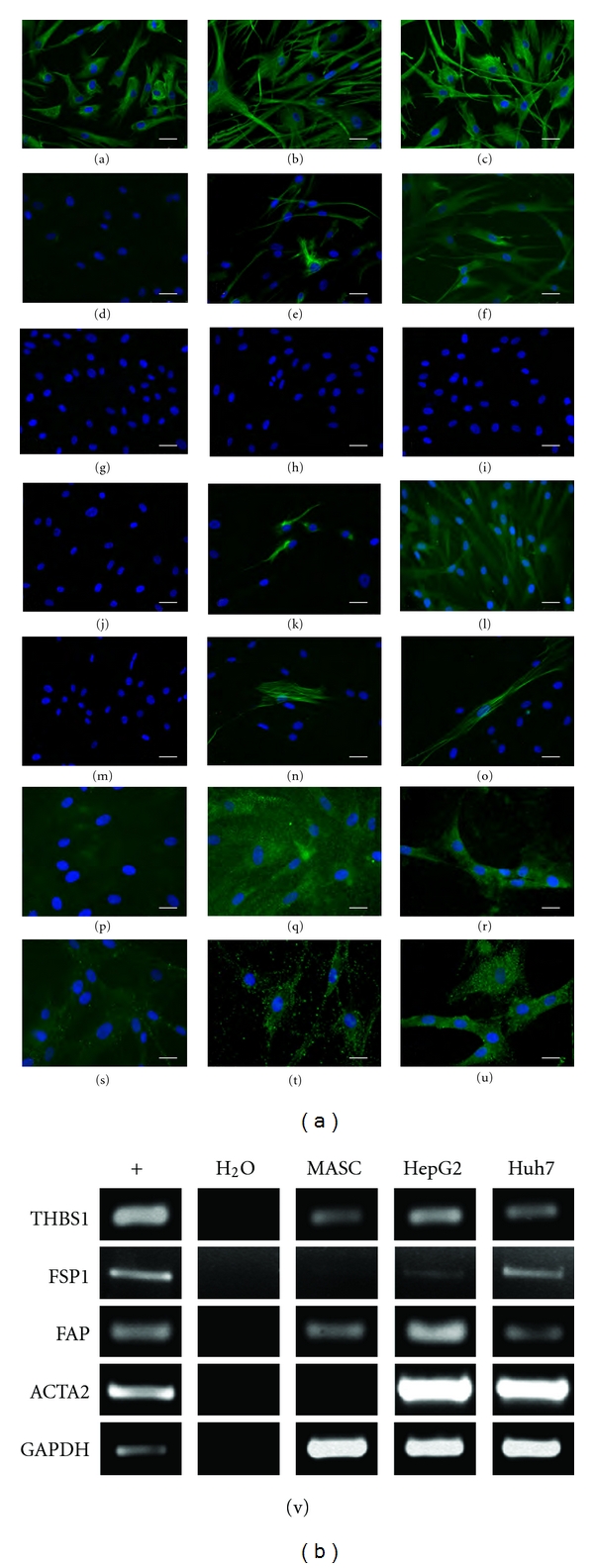
L-MASCs cultured in the presence of tumor-conditioned medium (CM) acquire an activated phenotype. (a–u) Protein expression of TAF-markers. L-MASCs cultured in non-CM (a, d, g, j, m, p, s), in HepG2-CM (b, e, h, k, n, q, t), and in Huh7-CM (c, f, i, l, o, r, u) were assessed for the expression of vimentin (green fluorescence, (a–c)), nestin (green fluorescence, (d–f)), cytokeratines (green fluorescence, (g–i)), desmin (green fluorescence, (j–l)), smooth muscle actin (green fluorescence, (m–o)), tenascin-C (p–r)), and VEGF (green fluorescence, (s–u)). Nuclei are depicted by the blue fluorescence of DAPI staining. Scale bar = 30 *μ*m (a–o), 20 *μ*m (p–u). (v) mRNA expression of TAF markers. Representative RT-PCR evaluating the expression of THBS1, FSP1, FAP, ACTA2, and GAPDH in L-MASCs cultured in non-CM, HepG2-CM, and Huh7-CM, respectively. HeLa was utilized as positive control (+).

Conversely, L-MASCs cultured in the presence of HepG2-CM or Huh7-CM, started to express a significant increased amount of TAF-specific markers such as desmin, smooth muscle actin, tenascin C and VEGF (Figures  5(a)–5(u), Table 3), fibroblast-specific protein (FSP), thrombospondin-1 (THBS1), and fibroblast activating protein (FAP) (Figure  5(v)).

**Figure 6:**
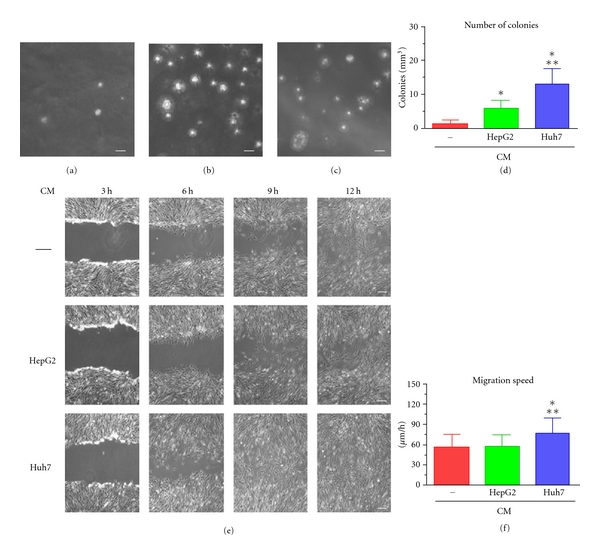
L-MASCs cultured in the presence of tumor-conditioned medium modify their growth characteristics. (a–d) Anchorage independent growth (soft agar assay). Phase contrast images of the colonies formed by L-MASCs grown in non-CM (a), HepG2-CM (b), and Huh7-CM (c). Scale bar = 100 *μ*m. Results are expressed as mean ± S.D. ^∗, ∗∗^ *P* < 0.05 versus non-CM and HepG2-CM, respectively (d). (e–f) Migration ability (scratch assay). Phase contrast images of three scratches produced in L-MASCs grown in non-CM, HepG2-CM, and Huh7-CM, respectively, taken at different time points. Scale bar = 50 *μ*m. Results are expressed as mean ± S.D. ^∗, ∗∗^ *P* < 0.05 versus non-CM and HepG2-CM, respectively (f).

Importantly, cell lines grown in the presence of either Huh7- or HepG2-CM displayed an at least 10-fold increased ability to grow in anchorage-independent conditions (Figures  6(a)–6(d)), while no significant differences in the mean colony size were seen among the three different culture conditions.

Moreover, evaluating the migration ability by scratch assay (Figure  6(e)), we established that L-MASCs grown in a medium conditioned by Huh7 displayed, with respect to L-MASCs grown in non-CM or HepG2-CM, a significantly increased migration speed (Figures  6(e)–6(f)). 

Altogether, these results showed that MASCs obtained from noncirrhotic and nonneoplastic livers, when cultured in the presence of a medium conditioned by tumor cells, acquired properties specific of tumor associated fibroblasts.

## 4. Discussion

Similarly to what we have previously shown for other normal human tissues [24, 25], it is possible to isolate a population of multipotent adult stem cells (MASCs) from either cirrhotic or neoplastic specimens. Although the *in vivo* counterpart of MASCs is still undefined [24, 25], this cell type shares some features with activated hepatic stellate cells [34, 35]. Recently, it has been hypothesized that hepatic stellate cells are indeed progenitor cells, expressing Oct-4 and markers of all the three germ layers, which are able to give rise, *in vitro*, to endothelial cells and hepatocytes [34, 35]. Moreover, a fate-mapping study showed that stellate cells could become oval cells when activated in liver injury, and that these cells participate in ductular proliferation [36]. All these evidences point to the presence of mesenchymal, widely multipotent cells in adult tissues, which can take part to regenerative as well as inflammatory and neoplastic processes [37]. 

In this paper, we documented for the first time that MASCs isolated from HCC and cirrhotic samples have the main characteristics of TAFs, as opposed to those isolated from noncirrhotic, nonneoplastic livers. This result is in line with what has been shown for other solid tumors such as breast, pancreas, and prostate cancer [12, 17]. The ways in which TAFs could act on tumor growth are numerous. TAFs are contractile cells, associated with blood vessels, and able to produce growth factors (HGF, TGF-*β*, EGF, bFGF, and IGF), cytokines, chemokines, and enzymes, to degrade the extracellular matrix, and to act as immunomodulating cells, thus increasing tumor growth by creating a microenvironment suitable for this process [12, 17, 18]. Moreover, the presence of TAFs in the tumor is associated with an increased metastatic potential and, in general, with a poor prognosis [12]. 

Interestingly, the TAF aberrant growth properties, displayed by N- and C-MASCs, could persist despite extensive culture; this feature has also been described for other tumors and has been ascribed to epigenetic mechanisms [38, 39].

Since many of the neoplastic livers were surgical treated after transarterial chemoembolization (TACE), it could be postulated that TACE could have altered the growth behavior of MASCs, but (1) C-MASCs, with similar characteristics to those obtained from neoplastic livers, were obtained also from cirrhotic not neoplastic livers that were never exposed to chemoembolization; (2) the effect that medium conditioned by hepatocellular carcinoma cell lines exerts on normal liver cell lines showed that factors released by neoplastic cells can influence the *in vitro* behavior of L-MASCs, never exposed to chemoembolization. 

Importantly, the fact that TAF-like cells could be isolated from cirrhotic, nonneoplastic livers, suggests that cirrhosis is characterized by a microenvironment possibly favoring HCC onset. In fact, most cases of hepatocellular carcinoma are linked to the presence of cirrhosis [5, 6]. Therefore, there is a great interest in understanding the mechanisms that determine the appearance of neoplastic transformation in cirrhotic livers in order to prevent the development of cancer, identify useful markers for early diagnosis, and introduce novel therapeutic approaches. For example, several studies have highlighted the key role played in these processes by the fibroblast activating protein (FAP) [40]. This protein, involved in the production/degradation of the extracellular matrix [41], is expressed in cirrhotic livers by hepatic stellate cells and myofibroblasts [42]. Interestingly, FAP is also expressed by TAFs and was found to play a key role in the biology of different tumors, including hepatocellular carcinoma [43]. 

However, many questions remain unanswered. For example, it would be extremely interesting to understand the mechanism through which chronic proinflammatory insults give rise to TAFs and whether it is possible to detect etiology-based differences (e.g., viral infections versus autoimmune versus toxicity). In our casistic, C-MASCs isolated from alcohol-related (*n* = 3) and from virus-related cirrhosis (*n* = 15) showed similar characteristics in terms of surface immunophenotype and aberrant growth properties. However, an in depth comparison of the different etiology would require a larger sampling. Similarly, it would be important to understand whether TAFs obtained from neoplastic but not cirrhotic livers are different from the TAFs obtained from cirrhotic and neoplastic livers. Preliminary results obtained in our laboratory indicate that TAFs can be obtained from HCC arose in noncirrhotic livers (*n* = 2, data not shown), but it is necessary to increase the sample size to assess possible significant differences.

Finally, we have shown that although L-MASCs do not possess TAF features, when cultured in the presence of medium conditioned by tumor cell lines, they acquired aberrant growth properties and the ability to produce specific TAF markers [11, 12, 18]. Regarding the origin of TAFs, four possible sources were envisioned [12, 18, 22]: epithelial-mesenchymal transition of the neoplastic cells (excluded by the absence of genetic alterations within the TAFs) [12], recruitment and activation of resident fibroblasts, recruitment of circulating/bone marrow-derived mesenchymal stem cells, and recruitment of resident stem cells. Recent studies have shown that mesenchymal stem cells derived from bone marrow can give rise to TAFs [18, 20, 44]. Here we show that TAFs could arise from a population of resident primitive cells with mesenchymal features. In any case, the possibility that TAFs could derive from resident or circulating mesenchymal stem cells opens a new scenario for understanding the biology of cancer and to identify new therapeutic targets. In fact, although the mechanisms that regulate TAFs and their accumulation in tumor sites are not yet clarified, it seems clear that blocking the activation of TAFs and their continuous communication with the cancer cells could, in conjunction with chemotherapy regimens, limit tumor progression and metastasis [9, 22, 45]. One possible advantage in targeting the stroma includes the fact that TAFs are not as genetically unstable as malignant cells, making it less likely the onset of resistance to chemotherapy drugs [21, 46]. Some attempts have already been done in this direction. For example, intratumoral injection of an FAP-activated protoxin produced significant lysis and growth inhibition of human breast and prostate cancer xenografts with minimal toxicity to the host animal [45], while genetic deletion and pharmacologic inhibition of FAP inhibited tumor growth in both mouse models of lung and colon cancer [47]. Accordingly, it has been constructed an oral DNA vaccine targeting FAP able to suppress, in vaccinated animals, primary tumor cell growth and metastasis of multidrug-resistant murine colon and breast carcinoma [48]. 

The hypothesis that liver TAFs could originate from a population of primitive mesenchymal stem cells supports the notion that tumor-supporting stroma derived from a hierarchical cellular system in which rare stem cells, such as multipotent adult stem cells (MASCs), would be responsible for forming and instructing all the elements of the supportive microenvironment. The fact that TAFs can originate from a resident population of multipotent adult stem cells with a mesenchymal immunophenotype could explain some of the crucial TAF features. In fact, MASCs are able to differentiate into many stromal cell types and, as previously shown, their gene expression profile is characterized by a molecular signature consisting of a module of overexpressing genes involved in extracellular matrix remodeling, in immunomodulation, and in the production of growth factors and cytokines [24]. Selectively targeting this population, blocking its activation or differentiation into TAFs may be an innovative therapeutic approach that could act upstream with respect to the proposed therapies. Last, the possibility to have *in vitro* human models in which it is possible to induce TAF formation starting from normal multipotent adult stem cells could help in identifying key molecular events that could become target of novel interventions.

## 5. Conclusions

In conclusion, the two main findings emerged from this work are as follows. (1) Cirrhotic livers, not yet neoplastic, already possess a population of multipotent adult stem cells with TAF properties; these multipotent cells can be propagated in culture without reversion of the activated phenotype, and thus they represent a possible tool for understanding the biological mechanisms underlying the neoplastic transformation in cirrhotic livers. (2) Multipotent adult stem cells isolated from healthy livers can acquire a TAF phenotype when grown in conditioned medium from tumor cell lines, suggesting that multipotent cells residing in the liver may represent a population that, once recruited and conditioned by tumor cells, contributes to the formation of stroma and, in turn, to the progression of tumor.

Both the depicted models can play in the future a role in understanding biological phenomena related to hepato-carcinogenesis and in identifying novel therapies aimed at interfering with the interaction between stroma and cancer cells.

## Supplementary Material

Supplementary Movie: Spontaneous intracellular calcium oscillation in MASC undergoing myocyte differentiation, as assessed by Fluo-4 imaging (green fluorescence).

## Figures and Tables

**Table 1:** Clinical-pathological characteristics, HCC: hepatocellular carcinoma, N.D.: not determined, pre-treatment: transarterial chemoembolization.

No. of HCC patients	15
Age-yr	
Mean	58
s.d.	7
No. of male sex (%)	14 (93)
No. of infection (%)	14 (93)
No. of HCV (%)	8 (53)
No. of HBV (%)	5 (33)
No. of HBV + HDV (%)	1 (7)
No. of alcohol use (%)	1 (7)
No. of Pretreatment (%)	13 (87)
Surgery	
No. of OLTx (%)	13 (87)
No. of Resection (%)	2 (13)
HCC histopathological grade	
No. of G1 (%)	0 (0)
No. of G2 (%)	7 (47)
No. of G3 (%)	5 (33)
No. of G4 (%)	2 (13)
No. of not defined (%)	1 (7)

No. of cirrhotic but nonneoplastic livers	**3**

Age-yr	
Mean	58
s.d.	3
No. of male sex (%)	100
No. of infection (%)	0 (0)
No. of HCV (%)	0 (0)
No. of HBV (%)	1 (33)
No. of HBV + HDV(%)	0 (0)
No. of alcohol use (%)	2 (67)

No. of normal livers	**5**

Age-yr	
Mean	n.d.
s.d.	n.d.
No. of male sex (%)	n.d.

**Table 2:** Surface immunophenotype of N-, C-, and L-MASCs. Bonferroni post test: *P < 0.05 C- versus L-MASCs; **P < 0.05 N- versus L-MASCs.

		MASCs		*P*
	N	C	L

CD49b	99.3 ± 1.6	98.4 ± 4.1	84 ± 14	0,0008^∗, ∗∗^
CD13	98.6 ± 2.8	97.8 ± 5.4	97 ± 7	n.s.
CD73	97 ± 5.7	98.3 ± 2.9	93 ± 10	n.s.
CD44	90 ± 21.84	92.2 ± 14.5	91 ± 17	n.s.
CD90	90.6 ± 16.4	92.2 ± 19.5	79 ± 20	n.s.
CD59	89.2 ± 23.8	98.2 ± 4.1	82 ± 9	n.s.
CD29	88.5 ± 15.4	91.1 ± 19.4	79 ± 15	n.s.
CD49a	86 ± 33.3	89 ± 10.3	70 ± 9	n.s.
CD105	84.6 ± 12.9	86.3 ± 21.5	75 ± 16	n.s.
CD49d	54.9 ± 43.7	46.2 ± 31.6	98 ± 2	0.008*
E-cadherin	4.39 ± 7.5	3.75 ± 9	n.d.	
CD10	37.7 ± 28.8	31.1 ± 33.4	8.2 ± 10.9	n.s.
N-cadherin	2.56 ± 5.3	2.15 ± 5.1	n.d.	
CD66e	0.41 ± 0.58	0.24 ± 0.51	1.4 ± 1.7	n.s.
CD271	0.22 ± 0.20	0.39 ± 0.65	0.52 ± 0.16	n.s.
CD45	0.19 ± 0.26	0.16 ± 0.35	0.05 ± 0.04	n.s.
ABCG2	0.18 ± 0.10	0.24 ± 0.2	0.21 ± 0.12	n.s.
CD117	0.17 ± 0.23	0.11 ± 0.15	0.03 ± 0.05	n.s.
CD133	0.17 ± 0.19	0.23 ± 0.27	0.18 ± 0.24	n.s.
CD34	0.11 ± 0.10	0.10 ± 0.18	0.03 ± 0.05	n.s.

**Table 3:** Quantification of the changes in the expression of specific markers in L-MASCs cultured in the presence of non-CM, HepG2-CM and HuH7-CM. Data are expressed as mean ± standard deviation. Neg = no expression.

	No-CM	HepG2-CM	HuH7-CM
Vimentin	99 ± 1%	97 ± 3%	97 ± 3%
Nestin	2 ± 1%	15 ± 15%	30 ± 5
Cytokeratins	Neg	Neg	Neg
Desmin	Neg	38 ± 5%	85 ± 10%
Smooth muscle actin	Neg	7 ± 8%	11 ± 6%
Tenascin-C	10 ± 2%	95 ± 3%	98 ± 2%
VEGF	4 ± 3%	70 ± 21%	85 ± 15%
